# Towards a Food Safety Knowledge Base Applicable in Crisis Situations and Beyond

**DOI:** 10.1155/2015/830809

**Published:** 2015-07-13

**Authors:** Alexander Falenski, Armin A. Weiser, Christian Thöns, Bernd Appel, Annemarie Käsbohrer, Matthias Filter

**Affiliations:** Federal Institute for Risk Assessment, Diedersdorfer Weg 1, 12277 Berlin, Germany

## Abstract

In case of contamination in the food chain, fast action is required in order to reduce the numbers of affected people. In such situations, being able to predict the fate of agents in foods would help risk assessors and decision makers in assessing the potential effects of a specific contamination event and thus enable them to deduce the appropriate mitigation measures. One efficient strategy supporting this is using model based simulations. However, application in crisis situations requires ready-to-use and easy-to-adapt models to be available from the so-called food safety knowledge bases. Here, we illustrate this concept and its benefits by applying the modular open source software tools PMM-Lab and FoodProcess-Lab. As a fictitious sample scenario, an intentional ricin contamination at a beef salami production facility was modelled. Predictive models describing the inactivation of ricin were reviewed, relevant models were implemented with PMM-Lab, and simulations on residual toxin amounts in the final product were performed with FoodProcess-Lab. Due to the generic and modular modelling concept implemented in these tools, they can be applied to simulate virtually any food safety contamination scenario. Apart from the application in crisis situations, the food safety knowledge base concept will also be useful in food quality and safety investigations.

## 1. Introduction

In pandemic crisis situations, timely and scientifically based exposure assessments are of crucial importance for all involved stakeholders (Regulation (EC) 178/2002) [1]. These exposure assessments are even more important in crisis scenarios in which the human population is at high risk. As could be witnessed in recent years during international foodborne disease outbreaks, tools and methodologies supporting efficient exposure assessments including the tracing back and forward of contaminated commodities are essential [2]. In an outbreak, risk assessors have to respond quickly to questions that crisis managers raise to support their decision-making processes. In order to empower risk assessors in their work predictive modelling software tools can provide valuable support, for example, by creating situation-specific models and running simulations for different contamination scenarios.

As a proof-of-principle scenario, the intentional contamination of a beef salami production facility with ricin was selected. Ricin is a toxin produced by the plant* Ricinus communis*. Its seeds are used for the production of over 1,100,000 tonnes of castor oil annually for various products, for example, paints, coatings, or polymers machines [3]. Because ricin is water soluble, it could be extracted from the remnants of the oil production for deliberate contamination of foods. The ricin content of the seeds makes up 1–5% [4, 5] or up to 1.5% in the castor cake after oil extraction [3, 6].

The toxin has been investigated in biological weapon programmes of the USA, Canada, and Iraq [7, 8]. It was presumably used in the assassination of the Bulgarian dissident Georgi Markov in 1978 [9] and was also found in a letter addressed to the White House in 2003 [10]. Ricin is classified as category B biological weapon [11].

The toxin acts as a protein synthesis inhibitor. In humans, the lethal oral dose is estimated to be 1–20 mg/kg body weight with symptoms like abdominal pain, vomiting, and diarrhoea [12]. Ricin is pH stable over a wide range [13]. For inactivation, high temperatures are needed. Pasteurization at 72°C for 15 seconds or at 89°C for 1 second did not fully inactivate ricin in infant milk formula [14]. Neither did the steaming of castor beans at 80°C for 40 minutes inactivate the toxin [15].

In the case of an intentional contamination of a food production chain, risk assessors need to estimate the consumers' exposure to the agent based on the amount of the contaminant that ends up in the final product. Additionally, the amount of agent introduced into the production chain needs to be estimated. Also, to be able to inactivate the agent in production machineries and intermediate products, it is necessary to know whether there are effective detergents for this purpose.

The aim of this research was to verify that a framework established for efficient and transparent conduction of exposure assessments in the food sector could also be applied in case of bio- and agroterroristic crisis situations. For this, data and models on tenacity of highly pathogenic agents were collected and applied in sample scenarios together with knowledge on relevant food production processes [16].

## 2. Material and Methods

### 2.1. Literature Review

A literature research on publications describing experimental data or models on the inactivation of ricin in food matrices was performed using the online databases PubMed (PM, http://www.ncbi.nlm.nih.gov/pubmed) and Web of Science (WoS, http://apps.webofknowledge.com) with standard settings. Publications were searched using the search string “ricin ((stability food) OR (inactivation food) OR beverage OR inactivation OR (models food) OR (predictive models)).” Database searches were performed in January and February 2014 according to the PRISMA statement for systematic reviews [17].

To gather information about the inactivation of ricin in foods, data and mathematical models were reviewed from the literature. All was stored in the internal database of the open-source modelling tool PMM-Lab (see Section 2.2).

Information on food processing chains was gathered from publications in scientific journals and from German text books about industrial and manual processing of beef or milk [18, 19]. Additionally, information on processing chains was collected via interviews of manufacturers and experts.

### 2.2. Food Safety Knowledgebase

The food safety knowledgebase consists of three components:

1. A collection of data and information on food production process parameters (FoodProcess knowledge base);
2. A collection of data and predictive models on relevant pathogenic agents (predictive model knowledge base);
3. A modular open-source software tool for exposure assessment calculations.

Technically, these components have been implemented in two modular software tools, PMM-Lab and FoodProcess-Lab (FPL). Both can be downloaded freely, installed locally, and used offline. This enables users to keep unpublished or confidential data on their desktop. The software also incorporates knowledge on food production processes and predictive models as well as the creation of scenarios as illustrated in Section 3.

PMM-Lab and FPL are both extensions to the scientific workflow management system KNIME (http://www.knime.org/) and inherit its modularity. The KNIME framework allows users to execute any data processing task by combination of small executable software modules, called nodes, into executable workflows. Each node's calculation result can be inspected visually at the node's outport. Plenty of nodes are available in KNIME and they can also be used from within PMM-Lab and FPL. In this way, it becomes possible to import data from all sources virtually and even to create reports automatically from workflows. In relation to food safety modelling, this modular workflow-based approach is therefore highly beneficial as the generation of prediction results becomes reproducible and transparently documented.

### 2.3. Software for Predictive Modelling (PMM-Lab)

PMM-Lab (http://sourceforge.net/projects/pmmlab) is a community resource for generation and application of predictive models, integrating more than 20 domain specific nodes as a new node library into KNIME. One of the advantages of this tool is that now raw data used to generate predictive models and the model generation workflow can be physically connected to the final model. This allows the data used for model generation to be viewed at any time. PMM-Lab additionally contains a database that stores all information (experimental data, models, metadata, and workflows) in relational database tables [20].

Additional information on how to use PMM-Lab can be found in the PMM-Lab Wiki (http://sourceforge.net/p/pmmlab/wiki/Home).

### 2.4. Software for Predicting the Tenacity of Pathogens along Food Processing Chains (FoodProcess-Lab)

FPL (http://sourceforge.net/projects/foodprocesslab) is like PMM-Lab, an extension to the KNIME framework providing six domain specific nodes structured inside the FPL node library. The “Ingredients” and “FoodProcess” nodes are the graphical representation of food processing chains and contain information on food processing parameters. All this information can be saved in the integrated database via the “Writer” node. Additionally, FPL can be used to perform mathematical calculations on agents spreading within food processing chains by the application of the “Agents” node. Via the “Filter Models” Tab of the “FoodProcess” node, the software can also make use of models on agent tenacity saved in the PMM-Lab section of the database. Information on food production process chains and parameters can be retrieved from the FPL database itself.

Finally, the “View” node can be used to graphically represent the change of food process conditions and agent concentration for the whole food process chain. Information about installation and sample workflows can be found in the FoodProcess-Lab Wiki (http://sourceforge.net/p/foodprocesslab/wiki/Home).

## 3. Results

Predictive models are important components of quantitative risk assessments. Several software tools exist which are designed to create predictive models based on pathogen specific experimental data (e.g., GinaFit [26], DMfit [27]). Additionally, there are tools allowing the application of models for predicting the tenacity of agents in different food matrices (e.g., ComBase Predictor [28], PMP [29], and SSSP [30]). However, in case of bio- and agroterrorist agents, authorities have to develop their own knowledge bases covering agent tenacity, considering typical food production processes including processing parameters. First efforts for a systematical collection of information concerning the latter aspect have been made in projects like FRISBEE (http://frisbee-wp2.chemeng.ntua.gr/coldchaindb) [31]. Unfortunately, the available solutions are currently only directed towards time-temperature profiles of postproduction processing steps. Additionally, models and software developed and used within the FRISBEE system are not freely available. In order to empower authorities to establish their own knowledge base in preparation for bio- or agroterrorist events free software access is highly beneficial. This requirement also takes into account the fact that in case of a real intentional food contamination situation the information exchange between authorities would be much easier if open-source software solutions already commonly used were applied.

### 3.1. Knowledge Base Generation

#### 3.1.1. Current Knowledge on the Inactivation of Ricin

**Figure 1:**
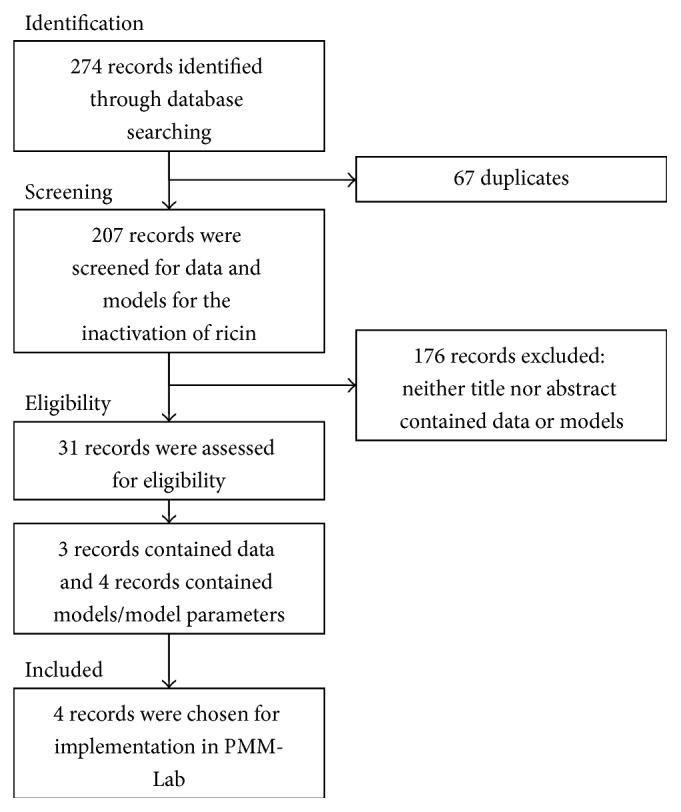
Flow diagram on the literature review performed precluding the implementation of models for the inactivation of ricin in foods with PMM-Lab.

In the literature research, a total of 274 entries dealing with the inactivation of ricin were retrieved from PubMed and Web of Science (see Figure 1). Of these, 31 publications were considered as relevant for in-depth analysis. Seven articles contained models, model parameters, or experimental data on the inactivation of ricin in food (see Table 1).

These papers describe the influence of temperature [14, 21–23], pH [13, 25], and chemicals [24] on ricin activity. Different food matrices as well as buffers were used in these reports: beef, milk, egg [22], infant formula [14, 24], orange juice, apple juice [21], pancake mix, peanut butter [24], phosphate buffered saline (PBS) [22, 24], sodium phosphate/sodium acetate buffer [13, 25], and KCl buffer [23]. The applied detection methods included fluorescence measurement, ELISA and cytotoxicity assays (see Table 1).

### 3.2. Ricin Model Repository

#### 3.2.1. Option 1 (Reimplementation of Models from Literature References)

Model equations, model parameters, and model metadata from the studies summarized in Table 1 were used to reimplement models using PMM-Lab. Model equations were entered as the so-called primary model formulas into the PMM-Lab Formula Creator node. Then, metadata and model parameters from the publications were copied to an MS Excel table and imported into PMM-Lab via the XLS Model Reader node. The generated primary models were saved to the local PMM-Lab model database.

#### 3.2.2. Option 2 (Model Estimation)

If not all model parameters necessary for reimplementation were given in a publication, proprietary models were created based on experimental data or parameter estimates in the publication. This approach was also applied, where parameter estimates were missing in published models. In case of the publication by Jackson et al. [21], the estimates on parameter “*A*” were not given (see (1)). In this case, the available primary models could be used to create proprietary secondary models allowing the inactivation of ricin to be predicted over the whole temperature range covered by the performed laboratory experiments. The so-called secondary models describe the relationship of primary model parameters to varying experimental conditions, like, for example, temperature. In this way, combined primary and secondary models can be used to interpolate ricin inactivation in the range of measured experimental conditions.

Jackson et al. [21] published a primary model formula which was fitted to measurements of residual ricin (1) after inactivation in different foods

(1) $$\begin{array}{c} \%ricin=Ae^{-kt}, \end{array}$$

where *A* is an empirically determined constant, *k* is the first-order rate constant, and *t* is the thermal treatment duration. As suggested in the publication, the Arrhenius equation (2) was then used as secondary model formula to create a model for the change of the first-order rate constant *k* with temperature

(2) $$\begin{array}{c} k=Be^{-E_{a}/\left(RT\right)}, \end{array}$$

where *B* is an empirically determined constant, *E*_*a*_ is the activation energy, *R* is the gas constant, and *T* is the temperature in Kelvin.

To simplify the parameter estimation process, the first-order rate constant was transformed by application of the natural logarithm transformation. Equations (1) and (2) were adjusted accordingly (see the following equation):

(3) %ricinAe−explnkt,lnk=lnB−EaRT.

Other equations were also tested for secondary model estimation, of which the second-order polynomial performed best as in the following equation:

(4) $$\begin{array}{c} ln\left(\;k\;\right)=a_{0}+a_{1}*T+a_{2}*T^{2}, \end{array}$$

where *a*_0_, *a*_1_, and *a*_2_ are empirically determined constants and *T* is the temperature in degrees Celsius.

**Figure 2:**
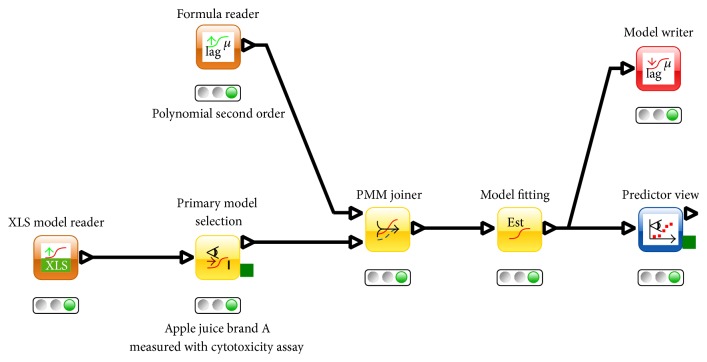
Estimation of proprietary secondary models and its application for prediction of ricin inactivation in apple juice.

The model generation workflow applied is depicted in Figure 2. As users can use the Formula Reader node to select equations from a wide formula collection implemented in the software, this very same workflow also allows alternative (better fitting) secondary models to be searched for. The results of the model fitting step performed with PMM-Lab are shown in Tables 2 and 3.

Overall, most of the fitted models had an *R* ^2^ greater than 0.94 with a few exceptions (minimum was 0.915). According to *R* ^2^, the polynomial models performed better than the Arrhenius-based models. In contrast, the AIC values from the Arrhenius models are lower than those from the polynomial models, indicating that the Arrhenius models should be preferred. This can be explained by the fact that Arrhenius models contain only two free model parameters instead of three in the polynomial models. As a consequence, the estimated Arrhenius-type secondary models were used to create a combined primary/secondary model which was saved into the PMM-Lab model database.

In comparison with the values for the inactivation energies published by Jackson et al. [21], half of those estimated with PMM-Lab match the published values (Table 2). However, four *E*_*a*_ values differ. In order to find higher agreement of published and estimated data, the original Arrhenius equation (non-ln-transformed) was used and one out of six outlying inactivation rates were omitted, resulting in a very high agreement with the published data (Table 2, last column; for full data, see Supplementary Table 3 in Supplementary Material available online at http://dx.doi.org/10.1155/2015/830809).

### 3.3. Food Process Model Repository

FPL can use knowledge on food processing chains stored in the integrated database. Furthermore, users can collect new information on processing parameters, for example, the processing steps for salami production. FPL also allows the user to describe processing chains with commodity flows that split up and join back again as in the case of the production of a meat product from carcasses (carcass → [processing to different pieces] → further processing, e.g., cutting or mincing → [addition of different parts of processed meat and fat] → meat product with standardized amount of fat). Values on temperature or other environmental conditions can either be entered as a single value, as a time-temperature profile, or as a function. The software also enables users to import knowledge on food production processes directly from other tools like CARVER [32].

**Figure 3:**
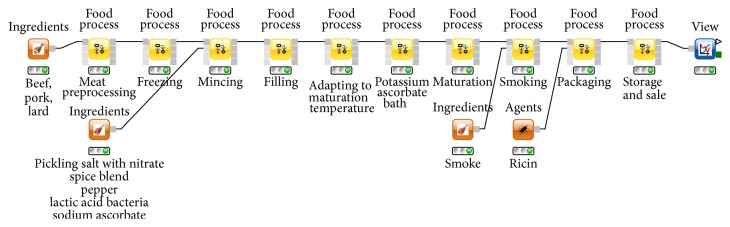
Process steps in the production of minisalamis as represented in FPL. Every yellow symbol (node) represents one processing step in the production of beef salami, configured in accordance with the data in Table 4.

As the basic design principle FPL represents each processing step as a food processing node (see Figure 3) which can be configured according to real world conditions. It is saved in the knowledge base or simply as a KNIME workflow. A similar modular concept was already introduced by Nauta [33, 34]. His Modular Process Risk Model (MPRM) is widely used in the domain of quantitative microbial risk assessments. In FPL, every FoodProcess node can both model microbial tenacity and calculate the effect of product handling changes. Its modularity refers to the reusability of single FoodProcess nodes as well as of full food processing chains (workflows). Together with the predictive models stored in the PMM-Lab database, the collected process information is then used as input for the predictions which are performed in each FPL process node [16].

### 3.4. Application of the Knowledge Base in Scenario Simulation

#### 3.4.1. Use Case 1 (Scenario Simulation)

In the following hypothetical contamination scenario, minisalamis (small salami sticks, weight: 10 g) are contaminated with ricin during their production. The food processing chain in brief is as follows [35]: Large parts of beef, pork, and lard are cut into pieces and are frozen before the mincing step (Table 4). The frozen meat is minced and pickling salt with nitrate, spices, sodium ascorbate, and lactic acid bacteria are added. The prepared meat is filled into casings and the raw sausages are warmed to room temperature. A short bath in potassium ascorbate prevents growth of bacteria on the surface of the casing. The minisalamis mature for 72 hours at 20–24°C and are smoked for 124 hours at 18–20°C. The hypothetical contamination of a production batch of the size of 100 kg with 3000 g pure ricin is set to take place during the process step “packaging,” meaning that the sausages would be contaminated on the outside. Finally, the minisalamis are stored and sold. The representation of the food processing chain in FPL and the introduction of ricin are both shown in Figure 3.

Ricin is a protein which degrades in the course of time, depending on temperature and pH. In the described scenario, the amount of active ricin was calculated with an inactivation rate published by Jackson et al. [21]. In the software, distinct predictive models can be assigned to each node/processing step. The amount and concentration of ricin left at the end of one node are “handed over” to the following node where it serves as the initial concentration.

**Figure 4:**
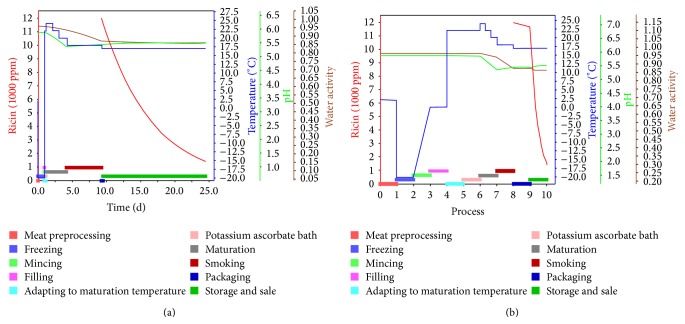
Visualisation of the change in processing parameters during the production of the minisalami. The coloured bars at the bottom of the graph show the different processing steps. The changes in temperature (blue), pH (green), and water activity (brown), as well as the inactivation curve of ricin (red), are shown as lines. The food processing chain is depicted as actual time series (a), for a clearer view on process steps, showing all process steps with equally wide bars (b).

For the given example, the time period for storage and disposal (until the customer is able to consume the product) was set to 15 days at 17°C.  Figure 4 shows the simulation results with respect to inactivation of ricin along the depicted processing chain. 12% of the introduced amount of ricin remains active until the day of consumption, resulting in a concentration of 4.3 mg ricin/g sausage. According to [12], the lethal oral dose for humans may be as low as 1 mg/kg of body weight. Thus, a person weighing 80 kg would already consume a deadly dose of ricin when eating only two minisalamis.

#### 3.4.2. Use Case 2 (Exploring Alternative Scenarios)

Contamination of a food processing chain can occur accidentally or intentionally and in various ways. In this example, ricin might not only be introduced at the packaging step, but also be mixed into the sausage meat during the mincing step or the toxin might be attached to the sausages during the maturation. In each of the three cases, the time until consumption is also an important factor concerning the amount of active ricin left. Therefore, the consequences of consuming minisalamis 7, 15, and 30 days after their production were calculated (Table 5). In most of the scenarios, the amount of active ricin left is below the lethal dose of 1 mg/kg of body weight. According to the model, this is the case when the salamis are contaminated during the mincing and maturation steps and if a minisalami contaminated during the packaging process was eaten a month later. In these cases, symptoms like nausea, vomiting, diarrhoea, and abdominal pain might occur [12]. On the other hand, if consumed only one week after production, each minisalami might still contain 131.5 mg active ricin which can lead to liver and renal dysfunction and death [12]. If salamis and packages were contaminated with ricin powder intoxication via inhalation is also possible. Depending on the particle size the lethal dose for inhalation ricin may be 50 times lower as was shown for monkeys [36].

## 4. Discussion

Models for the inactivation of ricin in foods were reviewed. Available models were implemented in PMM-Lab and used in a representation of a minisalami food process chain in FPL to predict the remaining amount of active ricin in the sausage.

### 4.1. Knowledge Base Generation

#### 4.1.1. Inactivation of Ricin

Only a minority of the publications contained information about the inactivation of ricin in foods. Mainly, the word “inactivation” led to abstracts about ribosomes inactivated by toxins. As ricin is a model toxin and representative for a whole group of ribosome inactivating toxins, many articles published findings about other toxins like shiga toxin or saporin, in which only the ricin toxin group was referenced. Finally, a considerable number of texts dealt with the molecular structure of ricin and other toxins. Publications were excluded if they did not contain information about ricin in foods in title or abstract. As a consequence, the uncertainty associated with the models applied in this research is quite high as the amount of independent experimental data that can be used for model generation is low. This underlines that in case of real bioterroristic crisis situations there will be a need for information exchange between governmental authorities which have more in-depth in-house data. Here, the open-source software framework can be supportive as well.

In case of new research areas about which no data are available it might be of help to use data and models about similar toxins as “proxy models.” This would not lead to exact predictions but might show into which direction research needs to go in order to find more quickly a solution.

#### 4.1.2. Ricin Model Repository

The works of William Tolleson and colleagues are truly a treasure for modellers when trying to calculate the inactivation of ricin in different foods. The published inactivation rates could be used as parameters in model formulas during the implementation of models in PMM-Lab. Unfortunately, not all parameters were noted, perhaps for safety reasons. For example, parameter *B* in (2) has not been published. This made the estimation of two unknowns (*B* and *E*_*a*_) necessary, leading to a greater variability. Of course, there was the possibility to use the published *E*_*a*_ values and simply recalculate all *B* values. But in this case the estimation of both parameters was chosen in order to estimate a secondary inactivation model with PMM-Lab. The activation energies (*E*_*a*_) shown above can only partly be called “in line” with those published by Jackson et al. [21].

Differences between *E*_*a*_ values might be a result of a different data basis: Jackson et al. [21] calculated the *E*_*a*_ using the original laboratory measurements and were able to derive the constant *B* empirically. In our case, both parameters were estimated applying the damped least square algorithm (also known as Levenberg-Marquardt algorithm) which is implemented in the PMM-Lab Model Fitting node. Due to these different parameter estimation approaches, the activation energies should not be used independent of the other parameter estimates.

Higher accordance with the published results could be obtained using the original Arrhenius equation. However, because of numerical reasons, a calculation of the covariance matrix was not possible with PMM-Lab.

#### 4.1.3. Food Process Model Repository

One of the advantages for the user working with the FPL plug-in is that no programming is necessary. Nodes can be dragged and dropped in order to create a new processing step. It is also a modular system, allowing the user to reuse workflows with new data or to copy parts of workflows as a basis for a new processing chain. In this way, different contamination scenarios can be easily visualized and worst case scenarios can be identified. Having information on whole food processing chains saved in the food safety knowledge base becomes most advantageous when an estimate on the fate of an agent has to be given quickly, as, for example, in crisis situations.

#### 4.1.4. Application of the Knowledge Base in Scenario Simulations

Above, several scenarios for the contamination of the minisalami food chain with ricin are described. Many more could easily be set up, for example, in order to consider food distribution in a more detailed way. However, data suitable for predictive modelling is frequently hard to find. In this case, a well-documented food processing chain containing slopes for temperature, pH value, and water activity was available. It could be directly implemented into FPL.

However, published inactivation rates for ricin in foods are scarce and mainly describe the thermal inactivation between 60 and 90°C [14, 21, 23, 24]. In the minisalami production chain, temperatures range from −20°C to 24°C, of which the most important process steps (maturation, smoking, storage, and sale) have temperatures between 17 and 24°C. Due to the lack of data, a rate for the inactivation of ricin in PBS at 25°C and pH 3.8 was used [21]. It was used in a primary model, meaning that changes in process temperature, pH, and water activity were not considered. For a suitable prediction of ricin inactivation, a secondary model for every interim product in the food chain would be necessary. As the model temperature is higher (25°C) than the food process temperatures (−20–24°C), this is not a fail-safe prediction and a higher proportion of ricin might remain active in the minisalami compared to the amounts calculated here.

However, the objective for generating scenarios in this paper was to show what is possible with publicly available data and published scientific literature. Risk assessors in the crisis and defence sector perhaps have access to unpublished data. The combination of PMM-Lab and FoodProcess-Lab is a powerful tool for which an easy-to-explain proof-of-principle example was provided.

The contamination of a food chain with a toxin is just one example. The growth of* Salmonella* during the production of accidentally contaminated minced chicken meat and the growth of* Listeria monocytogenes* in raw milk cheese are examples of bacterial contamination which can also be modelled with FPL and PMM-Lab, provided that necessary data and models are available.

#### 4.1.5. Knowledge Base Consolidation

The creation of food safety knowledge bases is not only a technical or scientific challenge. Successful building-up and consolidation require acceptance and support by experimental researchers, modelling experts, and end users. This implies that several related issues have to be addressed in parallel with the technical implementation of knowledge bases. First, an internationally harmonized data exchange format for information related to food safety modelling would be of enormous value. Such a data exchange format would allow scientists to report their experimental data or models in a standardized way independent from the software used. This would improve transparency and quality control significantly, as it then becomes possible to provide unambiguous information on the data sets used for model generation, for example. It would also support existing food safety data collections like ComBase (http://www.combase.cc/) as software tools could be developed (or extended) that support information exchange. A first proposal for such a food safety information exchange format has recently been published at the OpenML for Predictive Modelling in Food community portal: http://sourceforge.net/projects/microbialmodelingexchange/. Second, the idea of sharing data and models within the scientific community needs to be promoted. In this sense, it would be beneficial if the opportunity to provide experimental data and models as supplementary materials to scientific publications would be widely advocated. This would also support the establishment of supervised community knowledge bases. These resources could in turn assign persistent URIs to data sets or models which would allow them to be referenced directly. The third relevant issue for knowledge base consolidation is related to the presentation and visualization of modelling results to the end users. The challenge here is that the latter demand easy-to-interpret answers to questions that usually require complex modelling efforts. Additionally, model based predictions might differ depending on the model used. Currently available solutions for illustration of model related uncertainties still impair end users' understanding. Also, the heterogeneity of user interfaces in existing and emerging software tools is challenging for end users. Here, open-source software projects could help to disseminate solutions with high usability and functionality as these components could then be reused by other software developers in their tools.

## 5. Conclusions

With this work, a proof-of-principle for a food safety knowledgebase applicable in bioterroristic crisis scenarios is delivered. FPL as a free-community resource can be used to represent, save, and exchange food processing chains. In combination with PMM-Lab, the inactivation of toxins as well as the tenacity of bacteria can be modelled along these food chains, providing means for exposure assessment. Once a knowledge base is built up, this will be of great help in crisis situations and beyond.

## Supplementary Material

Tables 2 and 3 of the publication show parameter estimations based on all data published by Jackson et al. (2010). Leaving out data points can lead to a better fitting of the model which is shown in Supplementary Tables 1 and 2. Table 2 shows in the last column only the activation energies calculated using the original Arrhenius equation. To provide all parameter estimations, Supplementary Table 3 was added here.

## Figures and Tables

**Table 1:** Studies on inactivation of ricin in food matrices.

Matrix	Environmental conditions	Detection method(s)	Rate constants^a^	Reference
Apple juice	25°C; 60–90°C (*n* = 6)	ELISA, cytotoxicity assay	✓	[21]
Beef	63°C; 72°C	Fluorescence		[22]
Buffers (HCl, KCl, glycine, acetic acid, KOH, KH_2_PH_4_, K_2_HPO_4_, boric acid, KHCO_3_, K_2_CO_3_)	43.9, 52.9, 65.3, 71.5, 78.2, 86.5°C pH 1–12	Visible light (colour change)	✓	[23]
Egg	63°C; 72°C	Fluorescence		[22]
Infant formula	60–90°C (*n* = 6)	ELISA, cytotoxicity assay	✓	[14]
Infant formula^c^	NaClO (1.3, 6.7, 13 mM) at RT	ELISA, cytotoxicity assay	✓	[24]
Infant formula^c^	PAA (6.6, 13, 26 mM) at RT	ELISA, cytotoxicity assay	✓	[24]
Milk	63°C; 72°C	Fluorescence		[22]
Na-phosphate/Na-acetate buffer	pH 3–10 (*n* = 16) at 20°C	Fluorescence		[13]
Na-phosphate/Na-acetate buffer	pH 2–7 at 25°C (*n* = 15) and 60°C (*n* = 6); 5–70°C at pH 7.3 (*n* = 16), 4.7 (*n* = 15), 4.0 (*n* = 10) and 3.0 (*n* = 8)	Fluorescence^b^		[25]
Orange juice	25°C; 60–90°C (*n* = 6)	ELISA, cytotoxicity assay	✓	[21]
Pancake mix^c^	NaClO (6.7, 13, 27 mM) at RT	ELISA, cytotoxicity assay	✓	[24]
Pancake mix^c^	PAA (6.6, 13 mM) at RT	ELISA, cytotoxicity assay	✓	[24]
Pancake mix^c^	PAA-based disinfectant (1.0, 3.0, 5.0% with pH 5.0, 4.4, 3.9, resp.) at RT	ELISA	✓	[24]
Pancake mix^c^	CAD (3.0, 5.0, 7.0% with pH 10.2, 11.0, 12.1, resp.) at RT	ELISA	✓	[24]
PBS	63°C; 72°C	Fluorescence		[22]
PBS^c^	NaClO (67, 130, 270 *µ*M) at RT	ELISA, cytotoxicity assay	✓	[24]
PBS^c^	PAA (6.6, 13, 26 mM) at RT	ELISA, cytotoxicity assay	✓	[24]
PBS^c^	PAA-based disinfectant (0.1, 0.5, 1.0% with pH 6.2, 5.7, 5.0, resp.) at RT	ELISA	✓	[24]
PBS^c^	CAD (0.5, 2.0, 5.0% with pH 8.8, 9.7, 11.0, resp.) at RT	ELISA	✓	[24]
Peanut butter^c^	NaClO (13, 27, 40 mM) at RT	ELISA, cytotoxicity assay	✓	[24]
Peanut butter^c^	PAA (39, 66, 130 mM) at RT	ELISA, cytotoxicity assay	✓	[24]
Peanut butter^c^	PAA-based disinfectant (1.0, 3.0, 5.0% with pH 5.0, 4.4, 3.9, resp.) at RT	ELISA	✓	[24]
Peanut butter^c^	CAD (3.0, 5.0, 7.0% with pH 10.2, 11.0, 12.1, resp.) at RT	ELISA	✓	[24]

^a^✓: published rate constants useful for modelling the inactivation of ricin in foods.

^b^Fluorescence of ricin B-chain.

^c^In solution and dried on stainless steel coupons.

NaClO: sodium hypochlorite, CAD: chlorinated alkaline detergent, PAA: peracetic acid, and RT: room temperature.

**Table 2:** Secondary model estimation and quality criteria: Arrhenius equation, ln transformed; ln⁡(*k*) = ln⁡(*B*) − *E*_*a*_/(8.314∗*T*).

Matrix	Ricin inactivation measured with	*E*_*a*_ [kJ/mol] Jackson et al.	*E*_*a*_ [kJ/mol] This paper	ln⁡(*B*)	RMSE	*R* ^2^	AIC	*E*_*a*_ [kJ/mol] This paper^a^
Apple juice clear	ELISA	120 ± 10	188 ± 20	62.32	0.4891	0.9551	6.9853	118
Apple juice clear	Cytotoxicity assay	110 ± 20	185 ± 14	61.23	0.3448	0.9765	2.7898	119
Apple juice cloudy	ELISA	200 ± 11	187 ± 18	61.82	0.4298	0.9646	5.4350	204^b^
Apple juice cloudy	Cytotoxicity assay	240 ± 30	169 ± 18	55.95	0.4230	0.9582	5.2437	262^b^
Orange juice A	ELISA	170 ± 30	203 ± 31	67.47	0.7403	0.9154	11.9581	165
Orange juice A	Cytotoxicity assay	140 ± 30	177 ± 19	58.8	0.4579	0.9558	6.1937	139
Orange juice B	ELISA	170 ± 20	216 ± 28	72.1	0.6722	0.9370	10.8003	174^c^
Orange juice B	Cytotoxicity assay	161 ± 09	228 ± 26	76.23	0.6206	0.9511	9.8419	158

Values are estimated parameter values ± standard errors; ^a^Activation energies calculated using the original Arrhenius equation *k* = *B*∗exp⁡(−*E*_*a*_/(8.314∗*T*)); for a better fitting, some of the inactivation rates Jackson et al. [21] published were not used in this calculation (see ^b^ and ^c^); of the published inactivation rates at 60, 70, 75, 80, 85 and 90°C, the rate at 90°C was omitted in ^b^ and the rate at 85°C was omitted in ^c^. Standard errors could not be calculated. *T*: Temperature [K].

**Table 3:** Secondary model estimation and quality criteria: Polynomial of second order; ln⁡(*k*) = *a*_0_ + *a*_1_∗*T* + *a*_2_∗(*T* ^2^).

Matrix	Ricin inactivation measured with	*a*_0_	*a*_1_	*a*_2_	RMSE	*R* ^2^	AIC
Apple juice clear	ELISA	−37.5238	0.7551	−0.0038	0.3643	0.9813	31.7249
Apple juice clear	Cytotoxicity assay	−31.8713	0.5990	−0.0028	0.2646	0.9896	27.8857
Apple juice cloudy	ELISA	−23.2621	0.3635	−0.0012	0.4874	0.9659	35.2165
Apple juice cloudy	Cytotoxicity assay	−22.9993	0.3852	−0.0015	0.4703	0.9613	34.7882
Orange juice A	ELISA	3.0323	−0.3642	0.0038	0.6293	0.9541	38.2830
Orange juice A	Cytotoxicity assay	−6.3983	−0.0783	0.0017	0.4394	0.9695	33.9735
Orange juice B	ELISA	−10.0907	−0.0180	0.0016	0.7194	0.9459	39.8892
Orange juice B	Cytotoxicity assay	−25.3006	0.3829	−0.0010	0.7134	0.9515	39.7884

*T*: temperature [°C].

**Table 4:** Process steps in the production of beef salami.

Process step	Duration^a^	Temperature [°C]	Introduced ingredient	Ingredient mass [kg]	Product
Meat preprocessing	3 min	2	Raw beef Raw pork Lard	35 35 30	Pieces of raw beef, pork, lard
Freezing	24 h	−20			
Mincing	10 min	−20 → 0	Pickling salt with nitrate Spice blend Pepper Lactic acid bacteria Sodium ascorbate	2.8 1.3 0.3 0.08 0.05	Seasoned minced meat
Filling	3 min	0			Raw sausages
Adapting to maturation	4 h	22			
Potassium ascorbate bath	5 s	22			
Maturation	72 h	24 → 22 → 20			
Smoking	124 h	20 → 18	Smoke		Smoked minisalami
Packaging	4 h	17			
Storage and sale	7/15/34 d	17			

^a^Duration, d: day, h: hour, min: minute, and s: second.

**Table 5:** Contamination scenarios, calculated amount of active ricin left [mg] in minisalamis.

Process step	Day of consumption		
	7	15	30
Mincing	32.7	10.7	0.7
Maturation	33.5	10.9	0.8
Packaging	131.5	42.9	3.0
