# Supplementary material for: Towards a Food Safety Knowledge Base Applicable in Crisis Situations and Beyond
Source: Biomed Res Int. 2015 Jul 13;2015:830809. doi: 10.1155/2015/830809 (PMC4515494; doi:10.1155/2015/830809)
Supplement: Supplementary file 1 — Tables 2 and 3 of the publication show parameter estimations based on all data published by Jackson et al. (2010). Leaving out data points can lead to a better fitting of the model which is shown in Supplementary Tables 1 and 2. Table 2 shows in the last column only the activation energies calculated using the original Arrhenius equation. To provide all parameter estimations, Supplementary Table 3 was added here. [file 830809.f1.doc]

**Towards a food safety knowledge base applicable in crisis situations**

Falenski et al.

**Supplementary Information**

Tables 2 and 3 of the publication show parameter estimations based on all data published by Jackson et al. (2010). Leaving out data points can lead to a better fitting of the model which is shown in Supplementary Tables 1 and 2. Table 2 shows in the last column only the activation energies calculated using the original Arrhenius equation. To provide all parameter estimations, Supplementary Table 3 was added here.

**Supplementary Table 1:** Secondary model estimation and quality criteria: Polynomial of second order; ln (k) = a_0_ + a_1_ * T + a_2_ * (T^2^)

| **Matrix** | **Ricin inactivation measured with** | **a_0_** | **a_1_** | **a_2_** | **RMSE** | **R^2^** | **AIC** |
| --- | --- | --- | --- | --- | --- | --- | --- |
| Apple Juice clear | ELISA | -56.1540 | 1.2183 | -0.0067 | 0.3952 | 0.9527 | c.n.p.^a^ |
| Apple Juice clear | Cytotoxicity Assay | -32.4615 | 0.6332 | -0.0031 | 0.1473 | 0.9890 | c.n.p. ^a,b^ |
| Apple Juice cloudy | ELISA | -23.2621 | 0.3635 | -0.0012 | 0.4874 | 0.9659 | 35.2165 |
| Apple Juice cloudy | Cytotoxicity Assay | -16.8897 | 0.1706 | 0.0003 | 0.2930 | 0.9857 | c.n.p. ^a,c^ |
| Orange Juice A | ELISA | 3.0323 | -0.3642 | 0.0038 | 0.6293 | 0.9541 | 38.2830 |
| Orange Juice A | Cytotoxicity Assay | -6.3983 | -0.0783 | 0.0017 | 0.4394 | 0.9695 | 33.9735 |
| Orange Juice B | ELISA | -10.0907 | -0.0180 | 0.0016 | 0.7194 | 0.9459 | 39.8892 |
| Orange Juice B | Cytotoxicity Assay | -36.3589 | 0.7073 | -0.0033 | 0.0628 | 0.9988 | c.n.p. ^a,b^ |

For a better fitting, some of the inactivation rates Jackson et al. published were not used in this calculation; of the published inactivation rates at 60, 70, 75, 80, 85 and 90 °C, the rate at 60 °C was omitted in ^a)^, the rate at 70 °C was omitted in ^b)^ and the rate at 90 °C was omitted in ^c)^. T – Temperature [°C]; c.n.p. – calculation not possible.

**Supplementary Table 2:** Secondary model estimation and quality criteria: ln transformed Arrhenius equation; ln (k) = ln(B) – E_a_ / (8.314 * T)

| **Matrix** | **Ricin inactivation measured with** | **E_a_ [kJ/mol]**  **Jackson et al.** | **E_a_ [kJ/mol]**  **This paper** | **ln(B)** | **RMSE** | **R^2^** | **AIC** |
| --- | --- | --- | --- | --- | --- | --- | --- |
| Apple Juice clear | ELISA | 120 ± 10 | 160 ± 31 ^a^ | 52.8415 | 0.4656 | 0.9015 | 19.8009 |
| Apple Juice clear | Cytotoxicity Assay | 110 ± 20 | 130 ± 13 ^a,b^ | 42.7617 | 0.1423 | 0.9794 | c.n.p. |
| Apple Juice cloudy | ELISA | 200 ± 11 | 187 ± 18 | 61.8234 | 0.4298 | 0.9646 | 5.4350 |
| Apple Juice cloudy | Cytotoxicity Assay | 240 ± 30 | 222 ± 19 ^a,c^ | 74.2927 | 0.2095 | 0.9853 | c.n.p. |
| Orange Juice A | ELISA | 170 ± 30 | 203 ± 31 | 67.4662 | 0.7403 | 0.9154 | 11.9581 |
| Orange Juice A | Cytotoxicity Assay | 140 ± 30 | 177 ± 19 | 58.7997 | 0.4579 | 0.9558 | 6.1937 |
| Orange Juice B | ELISA | 170 ± 20 | 216 ± 28 | 72.0990 | 0.6722 | 0.9370 | 10.8003 |
| Orange Juice B | Cytotoxicity Assay | 161 ± 09 | 169 ± 10 ^a,b^ | 56.4780 | 0.1104 | 0.9925 | c.n.p. |

For a better fitting, some of the inactivation rates Jackson et al. published were not used in this calculation; of the published inactivation rates at 60, 70, 75, 80, 85 and 90 °C, the rate at 60 °C was omitted in ^a)^, the rate at 70 °C was omitted in ^b)^ and the rate at 90 °C was omitted in ^c)^. Values are estimated parameter values ± standard errors. T – Temperature [K] ; c.n.p. – calculation not possible.

**Supplementary Table 3:** Secondary model estimation and quality criteria: original Arrhenius equation; k=B*exp(-E_a_/(8.314*T))

| **Matrix** | **Ricin inactivation measured with** | **E_a_ [kJ/mol]**  **Jackson et al.** | **E_a_ [kJ/mol]**  **This paper** | **B** | **RMSE** | **R^2^** | **AIC** |
| --- | --- | --- | --- | --- | --- | --- | --- |
| Apple Juice clear | ELISA | 120 ± 10 | 118 | 6.46 x 10^16^ | 0.0451 | 0.9792 | -21.6256 |
| Apple Juice clear | Cytotoxicity Assay | 110 ± 20 | 119 | 7.87 x 10^16^ | 0.0443 | 0.9730 | -21.8365 |
| Apple Juice cloudy | ELISA | 200 ± 11 | 204 ^a^ | 2.93 x 10^29^ | 0.0121 | 0.9980 | -16.6659 |
| Apple Juice cloudy | Cytotoxicity Assay | 240 ± 30 | 262 ^a^ | 1.48 x 10^38^ | 0.0294 | 0.9948 | -7.8298 |
| Orange Juice A | ELISA | 170 ± 30 | 165 | 8.42 x 10^23^ | 0.1199 | 0.9699 | -9.8899 |
| Orange Juice A | Cytotoxicity Assay | 140 ± 30 | 139 | 1.22 x 10^20^ | 0.0973 | 0.9565 | -12.3934 |
| Orange Juice B | ELISA | 170 ± 20 | 174 ^b^ | 1.74 x 10^25^ | 0.0490 | 0.9948 | -2.7230 |
| Orange Juice B | Cytotoxicity Assay | 161 ± 09 | 158 | 8.94 x 10^22^ | 0.0407 | 0.9966 | -22.8366 |

For a better fitting, some of the inactivation rates Jackson et al. published were not used in this calculation; of the published inactivation rates at 60, 70, 75, 80, 85 and 90 °C, the rate at 90 °C was omitted in ^a)^ and the rate at 85 °C was omitted in ^b)^. Values are estimated parameter values ± standard errors. T – Temperature [K]
